# How do you perceive this author? Understanding and modeling authors’ communication quality in social media

**DOI:** 10.1371/journal.pone.0192061

**Published:** 2018-02-01

**Authors:** Kyungsik Han

**Affiliations:** Department of Software and Compute Engineering, Ajou University, Suwon, South Korea; Institut Català de Paleoecologia Humana i Evolució Social (IPHES), SPAIN

## Abstract

In this study, we leverage human evaluations, content analysis, and computational modeling to generate a comprehensive analysis of readers’ evaluations of authors’ communication quality in social media with respect to four factors: author credibility, interpersonal attraction, communication competence, and intent to interact. We review previous research on the human evaluation process and highlight its limitations in providing sufficient information for readers to assess authors’ communication quality. From our analysis of the evaluations of 1,000 Twitter authors’ communication quality from 300 human evaluators, we provide empirical evidence of the impact of the characteristics of the reader (demographic, social media experience, and personality), author (profile and social media engagement), and content (linguistic, syntactic, similarity, and sentiment) on the evaluation of an author’s communication quality. In addition, based on the author and message characteristics, we demonstrate the potential for building accurate models that can indicate an author’s communication quality.

## Introduction

The use of social media has been become a routine aspect of daily life that allows individuals to access and contribute to a wide range of information as well as to interact with many other users [1,2]. Despite its popularity for generating and disseminating information, there is growing concern about the reliability, trustworthiness, and credibility of social media information. False or misleading information (deceptions, rumors, propaganda, and half-truths) can easily and quickly be circulated to millions of users, who potentially can make wrong judgments and take inappropriate actions [3]. Therefore, it is important to detect spurious information and prevent it from running rampant throughout social media platforms.

However, from a reader’s standpoint, it is difficult to know or determine the credibility of social media information, given that every piece of information cannot be investigated. This generally leads to a situation where information credibility is determined by the readers themselves, based on their own intellect, experience, knowledge, and examination of other indicators [4,5]. Especially in social media, several heuristic cues, such as profile information, other posts, posting patterns, and level of engagement, are available to readers, from which readers can form their impressions, judgments, and perceptions of information credibility [6].

Research has adopted various approaches to investigate the credibility of social media information, frequently using Twitter as a platform. Those approaches have included characterizing credible and non-credible tweets, building machine-learning based classification models that differentiate non-credible tweets (or authors) from credible ones with high accuracy [7,8,9,10,11,12,13,14], and conducting user studies to investigate the impacts of factors that influence users’ credibility perception [15,16,17].

Although prior research has provided many insights regarding the reliability of social media information, our review of the literature indicates a few limitations with respect to the methodology and scope. First, the way of measuring information credibility is somewhat limited because only a single message or a set of random messages posted by different authors is given for credibility assessment; this is clearly not enough information (with a maximum of 140 characters) for evaluators to make a good decision [18]. This is presumably because preparing multiple messages by the same authors and presenting them to human evaluators makes the labeling process complex and time-intensive for both researchers and evaluators. Kumar and Geethakumari [3] highlighted that a reader’s cognitive process for evaluating information credibility is influenced by the consistency, coherency, and general acceptability of messages. It is thus important to provide evaluators with a sufficient number of author messages to ensure reliable responses.

Second, during the labeling process for obtaining ground-truth data for modeling, questions have been framed mostly to evaluate the credibility of the messages (e.g., “please rate the credibility of this tweet”), not the corresponding authors. Rather, the credibility of the authors was inferred based on the evaluations of their messages. Although content credibility is positively correlated to source credibility [19], readers’ evaluations of authors can be expanded and examined more comprehensively because readers can explicitly form impressions and evaluations of an author’s communication characteristics [20,21], which result from many factors specific to social media, including credibility, effectiveness of communication, writing style, and attractiveness of the author [22].

Third, untrue or manipulated content has often been used to measure the factors that influence readers’ perceptions of content credibility. While some control mechanism may be necessary to measure the influence of certain factors, using that type of content does not necessarily mirror reality.

In this paper, we address the above limitations and extend previous research efforts by designing a study to collect reader evaluations of authors from a series of actual, unedited posts from the authors. We obtain reader evaluations of authors based on their posts, and investigate various characteristics of the authors, their posts, and the corresponding *communication quality* [21], which includes *source credibility*, *interpersonal attraction*, *communication competence*, and *intent to interact*. In addition, we build classification models for determining whether authors can be characterized as having high or low communication quality.

We designed and framed our study based on the following research questions.

- **RQ1:** How do the linguistic characteristics of social media content and readers’ characteristics influence readers’ perceptions of an author’s communication quality?
- **RQ2:** What are the characteristics of social media content with respect to the degree of user agreement regarding an author’s communication quality?
- **RQ3:** Can we build models to predict an author’s communication quality with high accuracy?

To answer these research questions, we designed a user study with samples from a large corpus of tweets on Twitter. From our analysis of the evaluations of 1,000 Twitter authors’ communication quality from 300 human evaluators, this paper makes the following contributions:

- We present an in-depth analysis of an author’s communication quality in the social media context by leveraging various analytical approaches, including self-evaluation, content analysis, and computational modeling.
- We provide empirical evidence of the impact of various reader and content characteristics on assessments of an author’s communication quality.
- Based on these identified factors, we demonstrate the potential for building classification models to indicate the level of an author’s communication quality, with up to 94% accuracy (94% F1 score) when author, tweet, and linguistic features are used. Our study applies insights from social science to computational analyses.

To the best of our knowledge, this is the first study that has attempted to understand an author’s communication quality through large-scale, computational analyses, which extends the notion of credibility by leveraging human evaluations collected through a reliable and realistic scenario.

## Related work

### Information credibility in social media

Understanding credibility in social media contexts has long been studied. Our literature review indicates that existing research generally falls into two approaches: (1) identifying features and developing computational and prediction models, and (2) investigating the impact of various factors on one’s credibility perception through user studies.

#### Computational approach

Most machine-learning studies have used a method that identifies various features (e.g., from profile, content, topic, network, and affect) available in social media and obtain corresponding data to build computational models designed to classify credible and non-credible information. The way of obtaining the data in which information credibility is labeled has been done by asking humans to annotate the data or by defining behaviors of spammers (e.g., users whose accounts were suspended) [23]. Then researchers identify features for running the models. Some of such research efforts are as follows.

Ferrara et al. [9] found that features related to content, network, sentiment, and temporal patterns of activity are useful to detect computer bots compared to humans. Their study results indicated that bots tend to have more retweets and longer user names while human users tended to have a higher account age and more tweets. Agichtein et al. [7] presented content quality analysis in an online question and answer portal with focus on four elements. These included intrinsic content quality such as syntactic and semantic complexity (average number of syllables per word, word lengths, etc.), grammatical quality, user relationship (non-content information such as links between items and explicit quality ratings from members of the online community), and usage statistics (number of clicks on the item and dwell time). Their models showed the ability to separate high-quality items from the rest with an accuracy close to that of a human (precision and recall in the range of 80% to 95%). Lee, Caverlee, & Webb [24] proposed social honeypots as information system resources that monitor spammers’ behaviors and log their information (e.g., their profiles and other content created by them in social networking communities) and found that the best features for discriminating spammers and legitimate users are account age, and URL per tweet. Castillo et al. [8] identified four types of features including messages, users, topics, and propagation (the depth of the retweet tree) and presented the model results with precision and recall in the range of 70% to 85%. Yang et al. [14] identified graph- and network-based features such as bi-directional links ratio, between centrality, clustering coefficient, average neighbors’ followers, etc. and built models, which yielded 0.90 F1 score. Hentschel et al. [25] looked at the profile information of trusted users and found that the most commonly used profile images on Twitter are faces and 66% of the verified users have professional biographies.

In addition, research has focused on building credibility models in specific contexts (e.g., using the data generated during major events or incidents) where it would be more critical to have credible information. O’Donovan et al. [11] focused on tweets in emergency situations (e.g., earthquake), and used social, content, and behavioral features for modeling. They found that the best indicators of credibility include URLs, mentions, retweets, and tweet length—features that occur more prominently in data describing emergency and unrest situations. Gupta et al. [10] used an extensive set of 45 features (e.g., tweet meta-data features, tweet content features, user-based features, network features, linguistic features, and external resource features) for assessing the credibility of user-generated content on Twitter during six large scale emergencies (e.g., Boston Marathon Blasts and typhoons). Xia et al. [13] used 25 features from author, content, topic, and diffusion (e.g., time of tweet cited and time of the original tweets cited if they are retweets) from the tweets of UK Riots-related topics.

In summary, eliciting unique features and showing greater performance for their classification models (i.e., whether the information is credible or not) is one of the main research goals in computational research communities.

#### Human-centered approach

Many human-computer interaction studies have used a method that identifies underlying factors that may influence human’s (reader’s) credibility assessment. Unlike the computational approach, the human-centered approach has mostly employed surveys or interviews. Morris et al. [16] showed that the users are influenced by heuristics such as message topic, user name, and user profile image when making credibility assessments. Their user-study results indicated the impact of those heuristics in a single or combined fashion. For example, tweets on specific topics (news, political emergency, and consumers) caused the greatest concern about credibility. Altering the profile image made a greater difference in entertainment topics than in science or political topics. Similarly, Yang et al. [26] studied the impact of name style, profile image, location, and degree of network overlap with the reader on credibility perceptions and compared the results among US and Chinese audiences to see if there was a cultural influence as well. One of their findings indicates that people find tweets using photos for the profile image more credible than those using generic images, and there is a significant interaction between profile image and culture. As predicted, Chinese respondents are less sensitive to using anonymized images than US audiences.

Westerman et al. [5] examined the influence of the number of followers, follows, and the ratio between them on the reader’s perceived credibility. They found that authors who have too many or too few connections tended to be considered as less credible; and having a narrow gap between the number of followers and follows tended to increase the reader’s credibility perception. Lachlan et al. [27] investigated the relationship between the speed of updates on a Twitter feed and one’s perceptions of trust. Although there was no direct relationship found between the two, the authors found that one of the psychological factors, cognitive elaboration (i.e., the process of connecting newly obtained information with thoughts, memories, and scripts retrieved from personal experience), mediates the relationship. This study was extended by Spence et al. [28], where additional insights, namely the positive influence of the speed of updates on the perceptions of competence, goodwill, and trustworthiness, were presented.

Another interesting study for credibility assessment is the utilization of a specific score associated with it. Edwards et al. [29] used a Klout score, which is developed based on an idea that “everyone has influence—the ability to drive action.” A Klout score is computed based on three components: true reach (how many people a user influences), amplification (how much the user influences them), and network impact (the influence of the user’s network). Study results indicate that people perceived a Twitter page with a high Klout score more credible than a page with a low Klout score, demonstrating its influence and applicability.

While the studies primarily focused on the features available on Twitter, research has also investigated the readers’ credibility perception through the lenses of readers’ characteristics. Flanagin & Metzger [15] showed that men tended to rate credibility significantly higher than women. Shariff, Sanderson, & Zhang [17] presented a correlation analysis of readers’ demographics (e.g., gender, age, education, and location) and tweet credibility perception. They found that a reader’s educational background and geolocation have a significant correlation with credibility perception.

#### Limitations

The first limitation of a computational approach is that the method of obtaining human labels on information credibility is somewhat limited. In most cases, human annotators read a single, short (in case of Twitter, a single tweet consists of 140 characters), or random messages posted by multiple authors and were asked to rate the credibility of the message. Then the corresponding authors of those tweets are implicitly characterized based on the responses.

Credibility is a perceived quality and believability is composed of multiple dimensions [30]. Kumar & Geethakumari [3] explored the use of concepts from cognitive psychology to evaluate the spread of information and disinformation and showed that a reader’s cognitive process is influenced by the consistency of message, coherency of message, and general acceptability of message. Similarly, Kang [31] emphasized that the authority and reliability of the source and the focus of content are key aspects of social media credibility, and AlMansour et al. [32] indicate that source authority is one of the best predictors of credibility assessment. However, these aspects seem somewhat overlooked in the condition where user evaluations are done by accessing a single or multiple random message(s). Readers may not be able to make an accurate and reliable decision on information credibility from the limited amount of information, and it seems problematic to use many of their unclear responses to infer author’s credibility.

Our approach was to show enough tweets (10–15 tweets in our study) posted by the same author and ask readers’ perceptions towards the corresponding of the author of those tweets. Besides credibility, this author-based evaluation allows us to directly measure readers’ other perceptions, such as effectiveness of communication, writing style, attractiveness, etc., of the authors. Indeed, collecting one author’s multiple messages and presenting them to human evaluators makes a labeling process more complex and time-intensive, compared to the labeling process in prior studies. However, we believe this is a right way, because it allows the readers to see the overall style and patterns of the messages by the same author and to make accurate decisions.

A second limitation is that little research has been attempted that takes readers’ demographics into account. Only some research has considered limited information on the reader’s age, education, and geolocation [16]. Although research has conceptually highlighted that personality is positively related to trust [33] and traits and cognitive heuristics such as social media usage, demographics, propensity to trust, and personality [32] are important factors for credibility assessments on social media content; these traits lack empirical understandings and evaluations. We therefore addressed this by collecting responses from many readers and by measuring various reader characteristics in the study, and examined the relationships between those characteristics and communication quality evaluations.

Lastly, for the human-centered approach, some of the prior research used manipulated tweets to control their possible influence on an evaluator’s perceived credibility and measure the factors they tried to understand. This could lead to understanding credibility under unrealistic scenarios, and the manipulated examples with various controlled factors used in the study may not even exist on Twitter. To address this shortcoming, we used real and unedited tweets in our study, which allowed us to directly measure the relationships between linguistic characteristics of tweets and communication quality.

### Communication quality

Our goal is to understand the reader’s perceptions and evaluations of an author from the postings that the author has made. In this work, we employed a notion of a reader’s perceived communication quality studied in Edwards et al. [21], and modified the measures of each element that were more suitable for our study. Communication quality includes four elements:

- *Source credibility* refers to individuals’ perceptions of source credibility [34]. It was measured with respect to three dimensions (i.e., honest, trustworthy, and genuine).
- *Interpersonal attraction* refers to positive feelings about another person. It is not a unidimensional construct [35]. As studied in [21], we designed the measure of interpersonal attraction with respect to two dimensions: task (approving of and desiring interaction on an instrumental/goal-oriented level; two questions) and social (as liking and desiring affiliation on a relational level; two questions).
- *Communication competence* refers to a level of competence readers perceive the author to have in communication [36]. In our survey, it was measured by three questions that reflect an author’s ability of creating tweets with respect to clarity, effectiveness, and composure.
- *Intent to interact* refers to reader’s willingness to follow and obtain information from the author’s feeds. It was measured by two questions.

### Data collection and study design

**Fig 1:**
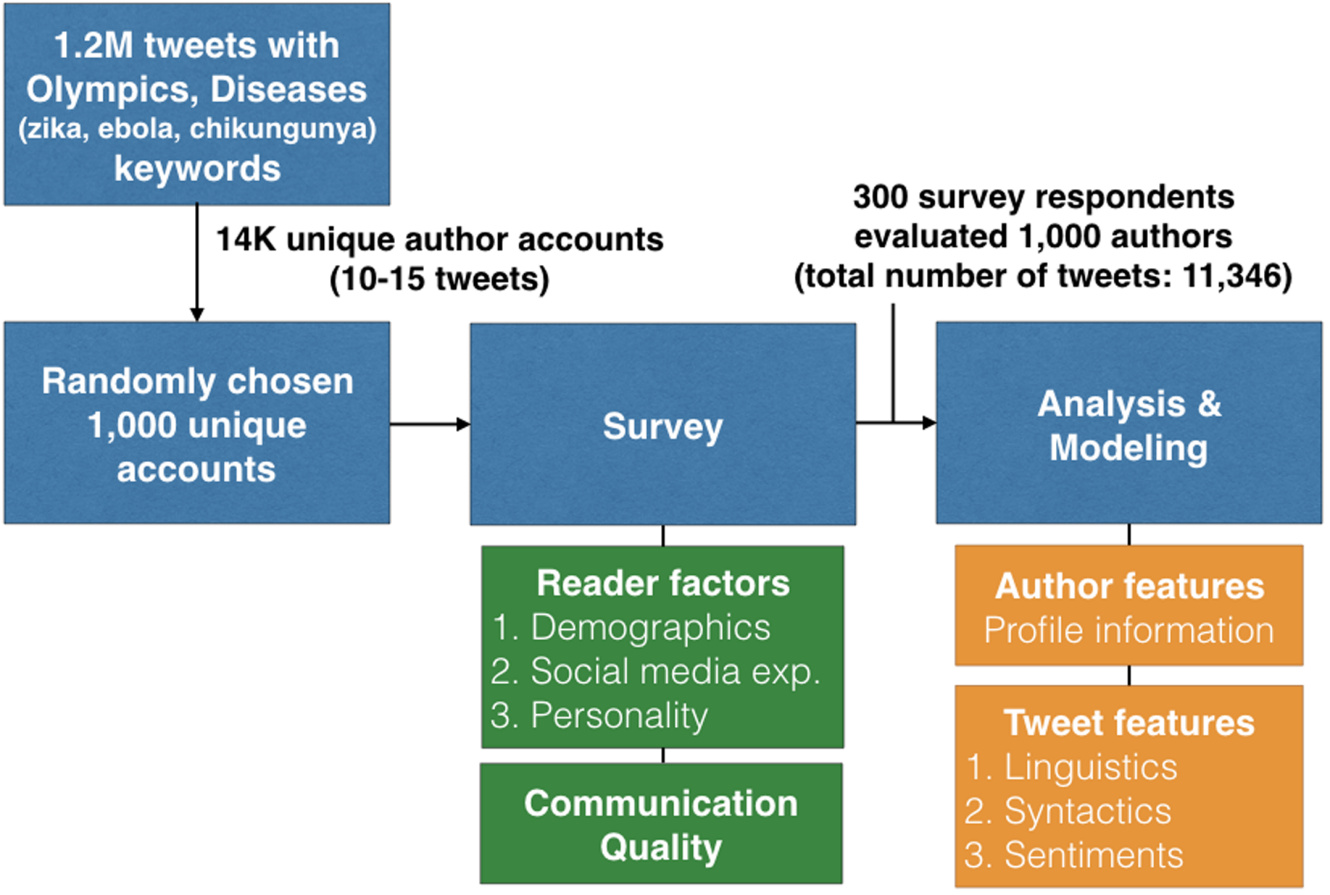
Overall study procedure.

The overall procedure of our research study is illustrated in Fig 1.

The rationale of our survey design is to use real tweets and present a set of tweets posted by the same author to give more information to the readers. We first collected a large corpus of tweets related to the 2016 Olympics (keyword: Olympics) posted between June and August of 2016 and three disease outbreaks (keyword: Zika, Ebola, Chikungunya) between January and April of 2017. We used the Twitter Search API (https://dev.twitter.com/rest/public) to collect tweets. As a result, we collected approximately 1,200,000 tweets written in English. From those tweets collected, we filtered out the authors who had fewer than ten tweets. After filtering, we had 15,260 authors. Lastly, we randomly chose 1,000 authors and their tweets and used them in the survey. Our intention to have a specific keyword (topic) in the tweet (rather than having any tweets posted by the same author) is that limiting the selection has a benefit for the readers in that the communication quality judgments may become easier to do since the sets are more easily comparable when topics are related.

Our study was reviewed and approved by the internal Institutional Review Board (IRB). We used SurveyMonkey and complied with its terms of service for data collection. None of the identifiable information of the survey respondents (e.g., names, account names, address, and phone number) was collected. As many studies have demonstrated the reliability and validity of Amazon Mechanical Turks [37], we used this service to collect responses. To ensure the quality of workers that we employed, we added a filtering mechanism that allowed us to recruit workers who have at least having 95% completion rates. The URL for the survey was included in the task. The respondents were first asked to read the informed consent that explains the goal and procedure of our survey study. Only the respondents who read and agreed with our study procedures and requirements could start answering the survey.

The survey consists of two main sections. In the first section, we asked respondents about their demographics, social media experience (use length and use frequency), and personalities, which were all presented with a 5-point Likert scale questions. We used the Big Five personality traits (extraversion, agreeableness, conscientiousness, neuroticism, and openness), a widely used model based on common language descriptors of personality [38]. In the second section, we asked respondents to read a set of tweets posted by the same author and to answer the questions about the author’s communication quality. Respondents read all tweets (in a random order to balance out fatigue and order effects) posted by the same author and were asked to judge at the same time. The instruments used for measuring communication quality are presented in Table 1.

**Table 1:** Instruments to measure communication quality. We used a 5-point Likert scale for each question (1: Strongly Disagree—5: Strongly Agree). Cronbach’s α results indicate high internal consistency among question items. In interpersonal attraction, the questions for IA1 and IA2 relate to social attraction and those for IA3 and IA4 relate to task attraction.

Type		Question (1: Strongly Disagree, 5: Strongly Agree)	α
**Source credibility (SC)**	SC1	The author seems honest	0.89
	SC2	The author seems trustworthy	
	SC3	The author seems genuine	
**Interpersonal attraction (IA)**	IA1	The author could be a friend of mine	0.92
	IA2	I could establish a personal friendship with the author	
	IA3	I have confidence in the author’s ability to get the job done	
	IA4	If I wanted to get things done, I could probably depend on the author	
**Communication competence (CC)**	CC1	The author seems effective in accomplishing what was set out to do	0.92
	CC2	The author’s tweets seem easy to understand	
	CC3	The author’s tweets seem written in a confident style	
**Intent to interact (INT)**	INT1	If the topic matches my interest, I may want to follow the author	0.93
	INT2	If the topic matches my interest, I may want to receive tweet updates from this author	

We ran a large-scale survey study. We prepared 20 surveys, where each survey has 50 authors with their tweets. We recruited a total of 300 respondents, and each respondent was assigned to one of the surveys. Thus, each author was evaluated by 15 survey respondents. The survey took approximately forty minutes to complete. For the statistical analysis, we used SPSS Statistics, and for the survey, we used SurveyMonkey. For both Twitter and SurveyMonkey, we complied with their terms of service for data collection.

## Results

After summarizing demographics of the survey respondents and the relationships among communication quality components, we present the relationships between the characteristics of the respondents (i.e., demographics, social media use, and personality) and communication quality. Next, we present the relationships between various linguistic characteristics of tweets and communication quality. Following that, we present the relationships between the author’s characteristics and communication quality. We then describe the characteristics of the author’s tweets that obtained low and high levels of consensus (agreement) among the respondents. Lastly, we present modeling of communication quality.

### Statistical analysis methods

We used three statistical analysis methods: Pearson’s correlation, multivariate linear regression, and analysis of variance (ANOVA). Especially for the multivariate linear regression, in order to measure the validity of the regression results, we additionally measured the following:

- *The Durbin Watson statistic test* is to measure autocorrelation in the residuals from a regression analysis [39]. The Durbin Watson statistic is always between 0 and 4. A value of 2 means that there is no autocorrelation in the sample. Values approaching 0 indicate positive autocorrelation and values toward 4 indicate negative autocorrelation.
- *The variance inflation factor (VIF) test* is to measure severity of multicollinearity in an ordinary least squares regression analysis [40]. It provides an index that measures how much the variance (the square of the estimate’s standard deviation) of an estimated regression coefficient is increased because of collinearity. The general rule of thumb is that VIFs exceeding 4 warrant further investigation, while VIFs exceeding 10 are signs of serious multicollinearity.
- We used the *stepwise linear regression* feature in SPSS. This allows to regress multiple independent variables while simultaneously removing those that are not important. It essentially goes in multiple regression a number of times, each time removing the weakest correlated variable. At the end, independent variables that best explain the distribution will be left.
- We also employed *hierarchical regression models* to see a specific relationship between independent variables that we are interested in and the dependent variables after controlling for other variables that may affect the relationship.

For the regression analysis, we report *R*^*2*^ and *F*-test results for the evaluation of model fit. In addition, before running the regression, we log-transformed all data for normalization.

### Survey respondents

Our survey respondents consisted of 179 (59.6%) males and 121 (40.4%) females; 198 (66.0%) respondents were in their twenties, 77 (25.6%) in their thirties, and 25 (8.4%) over forty years old. Among the respondents, 205 (68.3%) indicated that they have been using social media for more than four years, 69 (23.0%) between two to four years, and 26 (8.7%) fewer than two years. A total of 233 (77.6%) respondents indicated that they use social media at least once a day (Mean: 4.22, SD: 0.74, where 4-point means once a day and 5-point means several times a day).

### Communication quality of authors

Communication quality is the dependent variable in our study. As it includes four components, we looked at the relationship among those components. As shown in Table 2, all of them are closely correlated (p < 0.05), which complies with the study in [21]. As the four components are closely related as well as yield a very strong reliability result (Cronbach α = 0.97), and we aimed at measuring communication quality, combining them into one construct (we averaged them) and considering the construct as a degree of communication quality is a valid approach. With this central dependent variable, we were able to build classification models for a user’s communication quality.

**Table 2:** Correlations among four communication quality factors.

**Category**	**Credibility**	**Attraction**	**Competence**	**Intent**
Credibility	1.00			
Attraction	0.84*	1.00		
Competence	0.84*	0.86*	1.00	
Intent	0.76*	0.81*	0.76*	1.00

*p < 0.05

### Personal characteristics of readers and communication quality of authors

The Big Five personality traits include *extraversion* (level of sociability), *agreeableness* (level of friendliness and kindness to others), *conscientiousness* (level of organization and thoroughness), *neuroticism* (level of emotional stability), and *openness* (level of creativity and desire for knowledge and new experiences). With demographics and social media use, we measured the relationships among various aspects of personal information and communication quality.

We computed the relationships through multivariate linear regression. Table 3 (all independent variables) and Table 4 (step-wise regression) shows the standardized coefficients for four aspects of authors’ communication quality and author’s communication quality with personal characteristics. We found four interesting insights from this result.

**Table 3:** Standardized linear regression coefficients of authors’ communication quality with evaluators’ (readers’) gender, age, social media use length, frequency, and the five features of personality. The Durbin-Watson results are close to 2.0, showing that there is no presence of autocorrelation in the residuals. The VIF results for all predictors are less than 4.0.

Category	Credibility	Attraction	Competence	Intent	Comm Quality
Age	-0.102^†^	-0.144*	-0.090^†^	-0.175*	-0.134*
Gender	0.037	0.040	0.043	0.050	0.042
SM use length	0.024	-0.002	0.026	0.067	0.026
SM use frequency	0.135*	0.137*	0.166*	0.096^†^	0.146*
Extraversion	-0.006	-0.059	0.010	-0.021	-0.026
Agreeableness	0.053	0.057	0.076†	0.034	0.066†
Conscientiousness	-0.037	0.022	-0.018	-0.053	-0.018
Openness	0.004	0.041	0.001	-0.024	0.011
Neuroticism	-0.011	-0.041	-0.035	-0.020	-0.035
Durbin-Watson	1.979	1.988	2.001	2.048	2.059

^†^p < 0.10

*p < 0.05

**Table 4:** Stepwise regression result of Table 3. The Durbin-Watson results are close to 2.0, showing that there is no presence of autocorrelation in the residuals. The VIF results for all predictors are less than 4. Even we controlled for age, social media use frequency showed a significant influence on all components of communication quality and itself.

Category	Credibility	Attraction	Competence	Intent	Comm Quality
Age	-	-0.123*	-	-0.172*	-0.116*
SM use frequency	0.128*	0.121*	0.142*	-	0.121*
Durbin-Watson	1.979	1.988	2.001	2.048	2.059
*R*^*2*^	0.09	0.06	0.07	0.08	0.07
*F*	5.188*	5.389*	6.412*	9.531*	5.031*

*p < 0.05

First, results across the four communication quality components have variations to some extent. Second, age generally showed strong influence on communication quality, where the respondents in the older age group tended to perceive an author’s communication quality more negatively (conversely, those in the younger age group tended to perceive it more positively). Third, for gender, (although we did not obtain significant results) female respondents generally exhibited more positive perceptions than male respondents for all communication quality components. Fourth, it appears that frequent use of social media leads to having lower perception on author credibility and communication competence. The length of social media use did not show significant influence, but in general, other components and communication quality showed positive results except attraction.

Regarding personality, all traits did not show any strong effects. Yet, in general, agreeableness showed positive influence on communication competence and communication quality (at p < 0.10), including itself, whereas neuroticism showed negative influence overall.

Overall, these results demonstrate that a reader’s (human evaluator’s) perceived communication quality can vary depending on their own characteristics. Using human evaluations is a critical step for measuring both an author’s communication quality and information credibility. This insight emphasizes the importance of considering various reader factors to ensure consistency from evaluators.

### Tweet features and communication quality

Two methods were utilized to extract features from tweet messages. First, we used Linguistic Inquiry and Word Count (LIWC) [41], a text analysis program that captures style and content words as well as emotions in the document. Second, we measured additional features that are not available through LIWC but extractable in social media messages, including the number of hashtags, questions, exclamations, stocks, and emojis, sentiment scores, URLs, mentions, retweets, and tweets similarity. Tweets similarity was measured by transforming each tweet for the same author into TF-IDF vectors (vector size: 100), then computing the cosine similarity. This can only be measured through a study such as this, in which a set of tweets from the same author should be presented to and evaluated by humans.

We then computed multivariate regression on each of the four communication quality elements to see the relationships among LIWC and additional feature results, and communication quality. Tables 5 and 6 (step-wise regression) summarize the results, where we observed four noticeable insights.

**Table 5:** Standardized linear regression coefficients of authors’ communication quality with linguistic characteristics from LIWC and features unique in social media. The Durbin-Watson results are close to 2.0, showing that there is no presence of autocorrelation in the residuals. The VIF results for all predictors are less than 4.0.

Category	Credibility	Attraction	Competence	Intent	Comm Quality
Word count	-0.058	0.065	-0.001	0.008	0.001
Word per sentence	-0.020	-0.028	-0.040	-0.117*	-0.054
Sixltr words	0.153*	0.162*	0.152*	0.078†	0.155*
Dictionary	-0.068	-0.041	0.049	0.028	-0.040
1^st^ person singular	-0.038	-0.032	-0.021	-0.066	-0.046
1^st^ person plural	-0.022	-0.033	-0.074*	-0.021	-0.043
2^nd^ person	-0.023	-0.036	-0.025	-0.041	-0.036
3^rd^ person plural	-0.011	-0.025	-0.046	-0.005	-0.026
3^rd^ person overall	0.043	0.042	0.014	-0.007	0.031
Impersonal pronouns	0.017	0.044	0.037	0.015	0.034
Articles	0.126*	0.156*	0.107*	0.063†	0.117*
Auxiliary verbs	-0.045	-0.020	-0.054	0.079†	-0.020
Adverbs	-0.001	-0.032	0.003	0.056	0.000
Adjectives	0.013	0.016	0.037	0.013	0.022
Past tense	0.008	-0.007	-0.036	-0.012	-0.012
Present tense	0.036	0.075	0.030	-0.062	0.035
Future tense	0.024	0.044	0.021	0.014	0.032
Prepositions	0.124*	0.092*	0.079†	0.065†	0.106*
Conjunctions	0.061	0.077*	0.065†	-0.045	0.056
Negations	-0.056	-0.077†	-0.029	-0.089*	-0.071†
Numbers	0.073†	0.079*	0.056	-0.032	0.060
Quantifiers	0.087*	0.061†	0.048	-0.072*	0.047
Swear words	-0.067*	-0.014	-0.080	-0.005	-0.048
Average # hashtags	-0.091*	-0.043	-0.044	0.064	-0.042
Average # URLs	0.094†	0.053	0.073†	0.169*	0.079†
Average # mentions	-0.098*	-0.048	-0.086*	-0.115*	-0.093*
Average # retweets	-0.013	-0.022	-0.053	-0.010	-0.029
Average # questions	-0.071*	-0.112*	-0.074	-0.059†	-0.095*
Average # exclamations	-0.011	-0.016	-0.015	-0.008	-0.015
Average # stocks	-0.031	-0.001	-0.004	0.009	-0.009
Average # emojis	-0.043	-0.031	-0.030	0.026	-0.027
Average tweet length	0.001	-0.041	0.011	0.040	-0.002
Average sentiment	0.073*	0.069*	0.043	0.065*	0.068*
Tweets similarity	-0.170*	-0.146*	-0.152*	-0.166*	-0.179*
Durbin-Watson	1.434	1.353	1.434	1.208	1.401

^†^p < 0.10

*p < 0.05

**Table 6:** Stepwise regression result of Table 5. The VIF results for all predictors are less than 4.0.

Category	Credibility	Attraction	Competence	Intent	Comm Quality
Sixltr words	0.148*	0.139*	0.136*	-	0.140*
Articles	0.132*	0.142*	0.126*	-	0.120*
Average sentiment	0.080*	0.128*	-	0.133*	0.073*
Tweet similarity	-0.165*	-0.149*	-0.146*	-0.168*	-0.171*
Prepositions	0.128*	0.090*	0.111*	-	0.096*
Average # mentions	-0.124*	-0.085*	-0.134*	-0.088*	-0.125*
Swear words	-0.070*	-	-0.079*	-	-
Average # questions	-0.067*	-0.106*	-0.061*	-	-0.087*
Average # hashtags	-0.079*	-	-	-	-
Quantifiers	0.088*	0.067*	-	-	-
Average # URLs	-	-	-	0.144*	-
Negations	-	-	-	-0.086*	-
WPS	-	-	-	-0.114*	-
Durbin-Watson	1.428	1.314	1.422	1.210	1.415
*R*^*2*^	0.123	0.128	0.113	0.103	0.119
*F*	3.811*	3.976*	3.472*	3.124*	3.675*

*p < 0.05

First, having more sixltr words (percentage of all words longer than 6 letters) leads to having greater credibility, attraction, communication competence, and communication quality (intent did not show a significant result but still positive coefficient at p < 0.10). Given that using sixltr words implies the components related to education and social class in the text and indicative of more complex and formal language [42]. Tweets written more formally seem to positively influence on evaluations on authors’ communication quality.

Second, having more articles (e.g., a, an, the) are positively related to all components of communication quality (except intent; yet the result still showed a positive coefficient at p < 0.10; Table 6), which indicates that use of concrete nouns or interest in objects and things leads to greater communication quality. Similarly, more use of prepositions indicates that the message is more formally written [42].

Third, we observed strong linguistic influences of sentiments in the tweets on credibility, competence, and intent. In addition, while positive emotions in the message generated positive perceptions, negative emotions generally yielded negative perceptions.

Fourth, for the features of tweet messages, having more questions, mentions, tweets written in a similar fashion (at p < 0.05) and negations (at p < 0.10) typically lowered communication quality, while positive sentiments (at p < 0.05), more URLs and numbers (at p < 0.10) generally yielded positive influences on communication quality.

### Level of consensus

The evaluation criteria would differ depending on the respondents, which could affect the overall outcomes. It is therefore important to consider the degree of consensus (agreement) that respondents made toward the tweets used in the study. We expected that there might exist tweets that received similar evaluations as well as some that received high variations of evaluations. To measure consensus, we used the standard deviation of the mean scores of the evaluations, which we label σ (sigma). For this analysis, we considered the features of tweets (e.g., hashtags, sentiments, URLs, mentions, and retweets) as the independent variables.

Table 7 (all independent variables) and Table 8 (step-wise regression) summarizes the results after controlling for reader’s characteristics. A higher value means less consensus among the respondents. It appears that the similarity of the tweets strongly influences the level of consensus. In other words, readers’ evaluations tend to be less consistent over the tweets if the tweets look similar. Hashtags, mentions, and exclamations also showed relatively strong influence on the result.

**Table 7:** Standardized linear regression coefficients of communication quality with tweet features for the consensus analysis after controlling for reader’s characteristics. The Durbin-Watson results are close to 2.0, showing that there is no presence of autocorrelation in the residuals. The VIF results for all predictors are less than 4.0. Higher coefficient means less consensus among the respondents.

Category	Credibility	Attraction	Competence	Intent	Comm Quality
Average # hashtags	0.043	0.055†	0.025	0.099*	0.058†
Average # URLs	0.036	0.028	0.023	0.059	0.018
Average # mentions	0.088*	0.089*	0.078*	0.079*	0.093*
Average # retweets	0.022	0.026	0.027	0.056†	0.034
Average # questions	0.038	0.030	0.020	0.012	0.018
Average # exclamations	0.070	0.107*	0.087*	0.071*	0.095*
Average # stocks	0.013	0.024	0.029	0.040	0.028
Average # emojis	0.023	0.017	0.024	0.057†	0.030
Average sentiment	0.052†	0.094*	0.076*	0.069*	0.082*
Tweets similarity	0.213*	0.224*	0.216*	0.198*	0.236*
Durbin-Watson	1.200	1.145	1.248	1.094	1.042

^†^p < 0.10

*p < 0.05

**Table 8:** Stepwise regression result of Table 7. The VIF results for all predictors are less than 4.0.

Category	Credibility	Attraction	Competence	Intent	Comm Quality
Average # hashtags	-	-	-	0.115*	-
Average # mentions	0.092*	0.081*	0.093*	0.105*	0.109*
Average # exclamations	0.089*	0.111*	0.092*	0.067*	0.110*
Average sentiment	0.089*	0.095*	0.074*	0.071*	0.082*
Tweets similarity	0.214*	0.210*	0.208*	0.182*	0.225*
Durbin-Watson	1.200	1.145	1.248	1.094	1.042
*R*^*2*^	0.07	0.08	0.08	0.07	0.07
*F*	15.43*	20.94*	14.82*	13.74*	18.07*

*p < 0.05

We were then interested in the characteristics of the tweets that had high or low consensus. To do this, we first averaged four components of communication quality and sorted the results. We explored the tweets in the high score group (low consensus) and the low score group (high consensus).

**Fig 2:**
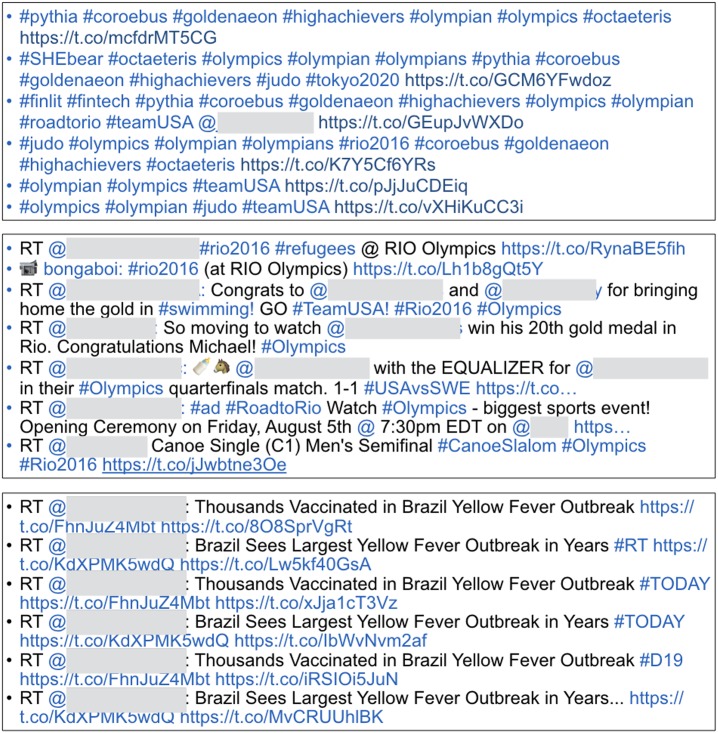
Three examples of tweets that yielded overall lowest consensus (high disagreement). First example (top) shows the tweets consisting of only hashtags and links. Second example (mid) shows the tweets with a high similarity score, mentions, and hashtags. Third example (bottom) shows the tweets that were written similarly. User identifiable information (mentions) is anonymized.

Fig 2 presents three examples of the tweets with highest disagreement. The first example shows a set of tweets consisting of many hashtags (p < 0.10). Its overall communication quality score was relatively low (mean: 2.59), and the average σ was above 1.0. This means that many respondents gave low scores but a few respondents granted high scores. Similarly, the second example shows a set of tweets with a high similarity score that contain mentions and hashtags. Like the first example, these tweets also received a low score (2.82), and the average σ was above 1.3, showing some degree of variations. The third example shows the tweets that look similar and seem to be redundant. The overall score in this case was 2.98, and the average σ was above 1.2. Having similar tweets for the same topic might negatively affect some of the readers’ scores. Overall, all of these are well aligned with the results in Tables 7 and 8.

**Fig 3:**
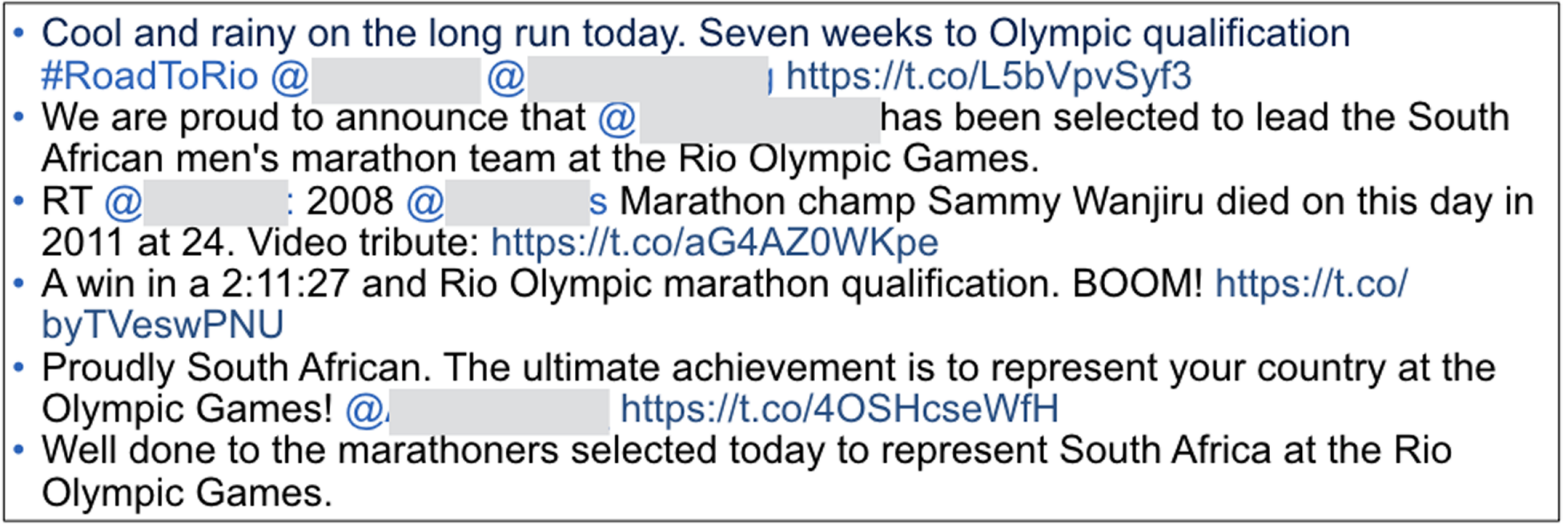
Example of tweets that yielded overall highest consensus (high agreement).

On the other hand, Fig 3 shows an example of the tweets that yielded the highest agreement (with overall score 3.75, and the average σ was 0.55). It appears that the respondents showed more agreement from the tweets that are not filled with many hashtags or mentions. This also well aligns with the results in Tables 7 and 8.

The findings and insights in this section emphasize the importance of taking various reader and content factors into account when measuring a reader’s perceived communication quality of an author based on his or her social media content.

### Modeling communication quality

We investigated the possibility of building reasonably accurate prediction models for characterizing tweets and authors with respect to communication quality.

We aimed at differentiating two author groups: one consisting of authors with high communication quality and one with low quality. To do this, we re-defined communication quality from a continuous variable to a categorical one. We used a 5-point Likert scale (1: Strongly disagree, 2: Disagree, 3: Neural, 4: Agree, and 5: Strongly Agree) in the survey, and with this range, we can assume that the score below 2.5 implies respondents’ relatively negative reactions and above 3.5 implies respondents’ positive reactions toward the authors. Thus, we defined the *high communication quality group* with an overall rating greater than 3.5, and the *low communication group* with a rating less than 2.5. This also places a significant gap between the two groups where 1-point difference at a 5-point Likert scale accounts for 20% difference. The t-test result shows a significant difference between the two groups: *t*(505) = 62.86 at p < 0.0001.

As a result, we had 91 authors in the low group and 416 authors in the high group. To have the same number of authors in the both groups, we used a popular re-sampling technique, SMOTETomek [43], which combines over-sampling and under-sampling methods. The Synthetic Minority Over-sampling Technique (SMOTE) algorithm applies KNN approach where it selects K nearest its neighbors, joins them and creates the synthetic samples in the space. Tomek links (TL) are used to remove examples after applying SMOTE, which are considered as noisy or lying in the decision border. Before running SMOTETomek, we first excluded the sample to use as a validation set, and then oversampled the remaining of the minority class (low group). This is a critical step, as the same data should not be used for training and validation, which will result in overfitting the models (http://www.marcoaltini.com/blog/dealing-with-imbalanced-data-undersampling-oversampling-and-proper-cross-validation). In this regard, we sampled 20% of our data for the validation data and applied SMOTE for the rest of the data. As a result, we built classification models based on 668 authors, and used 160 authors as the validation data.

#### Feature selection

For the features used in modeling, we used the common features related to authors (e.g., profile and engagement) and tweets (e.g., tweet syntactic, similarity, sentiments, and linguistics), which have been popularly used by many prior research studies on information credibility [7,8,9,10,11,13,23,24] (see the related work section for details). Using those well-known features, we wanted to build models that determine a level of communication quality. We removed all stop-words in the tweets and used the scikit-learn library (http://scikit-learn.org/stable/) for the analysis.

- **Author profile:** We used username, user profile description, registration age (day), and other usage reports (i.e., number of followers, follows, posts, likes, and lists) accessible in the user’s profile page.
- **Tweet syntactic:** We used all types of accessible information presented in a user’s tweet page including number of retweets, likes, question marks, exclamations, stock symbols, URLs, hashtags, and tweet word count and length.
- **Tweets similarity:** We used topic similarity that is measured by transforming each tweet for the same author into TF-IDF vectors [44], then computing the cosine similarity [45]. Compared to other features, tweet similarity has relatively less explored [24], as it asks for the collection of multiple tweets per author.
- **Tweet sentiments:** We used TextBlob (https://textblob.readthedocs.io/en/dev/quickstart.html) to calculate the sentiment score. It returns a level of polarity which is a float within the range [-1.0, 1.0], where -1.0 is very negative and 1.0 is very positive.
- **Tweet linguistics:** We used all linguistics characteristics (LIWC features).

#### Classification results

After collecting all features, we analyzed the data with different classification methods. We applied four widely adopted supervised learning models: Logistic Regression (LR), Support Vector Machine (SVM), Random Forest (RF), and Adaptive boosted Decision Trees (ADT).

For some features that are highly skewed, we pre-processed them through normalization in the range between 0 and 1, which reduces the side-effect of a long-tail distribution. We evaluated all the models using 5-fold cross validation and model performance through results of the accuracy, precision, recall, and F1 score. In addition, we used two common metrics for our evaluation of classification accuracy: area under the precision-recall curve (AUC), which measures how well each method can distinguish between high and low communication quality groups, and area under the receiver operating characteristic (ROC) curve, which measures the false positive rate required to experience a particular true positive rate. For the precision-recall curves, each algorithm assigns a score to each sample in the data set, where a higher score indicates that the sample presents better communication quality.

Table 9 shows the performance (accuracy, precision, recall, and F1 measure) of the four classification models with respect to three cases of feature usage: tweet features only, author features only, and all features. With all features included, we found that RF, which is well regarded as ensemble methods, yielded the best performance.

**Table 9:** Summary of accuracy, precision, recall, and F1 scores of the models based on different types of the features. Models with the tweet features yielded better performance than those with the author features. When all features were used, the models showed the best. Best model for each case is highlighted.

Feature type	Model	Accuracy	Precision	Recall	F1
**Tweet features only**	LR	0.74	0.75	0.74	0.74
	RF	0.92	0.92	0.92	0.92
	SVM	0.90	0.90	0.91	0.90
	ADT	0.90	0.90	0.91	0.91
**Author features only**	LR	0.71	0.71	0.72	0.72
	RF	0.84	0.84	0.84	0.84
	SVM	0.78	0.82	0.78	0.78
	ADT	0.83	0.84	0.83	0.84
**All features**	LR	0.76	0.76	0.75	0.76
	RF	0.94	0.95	0.93	0.94
	SVM	0.94	0.94	0.93	0.93
	ADT	0.94	0.94	0.93	0.93

**Fig 4:**
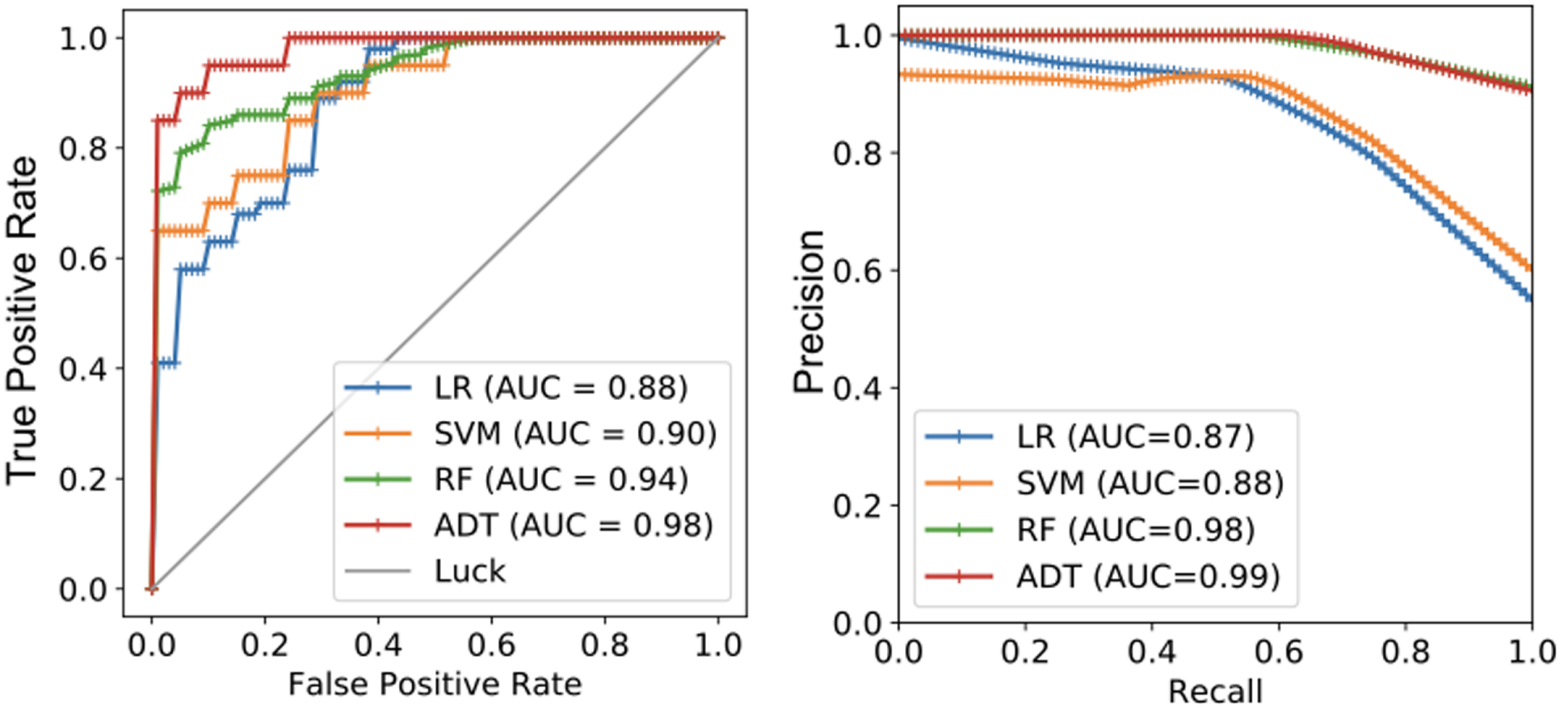
Result of ROC curve (left) and Precision-Recall Curve (right). RF and ADT yielded much greater performance than LR and SVM.

Fig 4 illustrates the results of the ROC curve and precision-recall curve. Overall, RF and ADT exhibited good results indicating that the features, which can be readily extracted and applied to any models, are useful in identifying the high quality communication and the low quality communication groups.

Note that our intention of building classification models is not to directly compare the performance our models with the ones in previous studies, because not only the dependent variables are different not also the labeling process is completely different (we asked the respondents to directly evaluate “authors” based on their tweets, whereas many prior studies focused on tweets). If it is assumed that it is fair to compare communication quality and credibility prediction results to get a very rough comparison (different data sets and labels), the performance of our models (0.94 F1 score) is greater than with the ones in previous studies, for example, 0.85 F1 score [46] and 0.86 F1 score [8] in classifying spammers and non-spammers.

**Fig 5:**
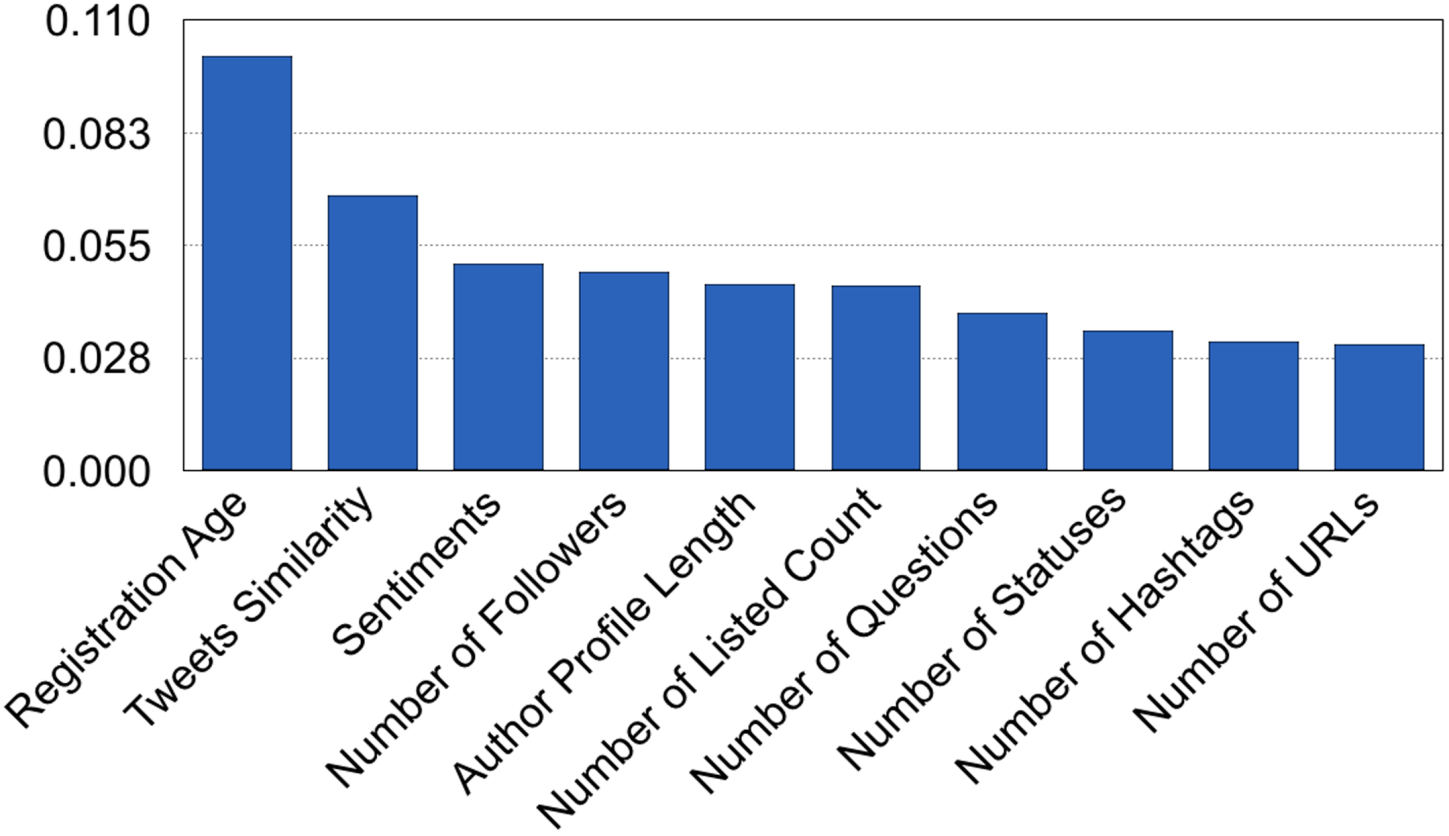
Top ten important features from the RF model.

As Random Forest showed the best performance, we further investigated the importance of the features used in this model. Fig 5 illustrates the use of forests of trees to evaluate the importance of features on a classification task. The blue bars are the feature importance of the forest, along with their inter-trees variability. We found that account registration age showed the greatest influence on the model, followed by tweets similarity, sentiments in the tweet.

In addition, we further investigated the statistical difference of the features between the high and low groups. Table 10 presents ten features that showed a significant difference between the two groups with respect to authors and tweets. Due to non-normalized datasets (showing high variances), we used eta-square (the effect size), denoted as η^2^, which refers to the proportion of variance associated with each of the main effects, interactions, and error in an ANOVA study [47]. As a rule, η^2^ = 0.01, 0.13, and 0.26 are small, medium, and large respectively.

**Table 10:** Author- and tweet-based feature comparison between the high and the low communication quality groups at p < 0.05 (sorted by F-values).

Type	Feature	Low	High	t(505)
Author	Author Registration Age (days)	1694.86	2357.09	40.57
	Author # favorites	18505.79	495127	9.37
Tweet	Tweet similarity	0.49	0.32	46.02
	Articles	2.01	3.07	18.29
	1st person singular	1.49	0.59	11.84
	Prepositions	7.97	9.26	11.02
	Sixltr words	23.36	25.91	10.53
	Swear words	0.42	0.11	8.09
	Average # questions	0.13	0.06	5.02
	Negations	0.79	0.53	4.26

Regarding author-based features, the high group tended to have longer account registration age (η^2^ = 0.14), but less number of favorites (η^2^ = 0.08). Regarding tweet-based features, compared to the low group, high group tended to have a low similarity among tweets (i.e., more diversity in describing keyword-related information; η^2^ = 0.06), more articles and preposition (i.e., the tweets were more objectively written, η^2^ = 0.07 and η^2^ = 0.08, respectively), more sixltr words (i.e., tweets were more formally written, η^2^ = 0.05), less swear words (η^2^ = 0.07), less 1^st^ person singular (i.e., tweets were more informally written, η^2^ = 0.04), less average number of questions (η^2^ = 0.09), and less negations (η^2^ = 0.05) in the tweets.

## Discussion

### Study summary and implications

We have presented a comprehensive analysis of readers’ evaluations of authors’ communication quality in social media through four lenses: author credibility, interpersonal attraction, communication competence, and intent to interact. In a methodological sense, our study provides unique insights, as we directly asked the readers to evaluate the authors by presenting a collection of tweets (more than 10 tweets) posted by the same authors, providing an author’s posting behaviors to the evaluators. Through the study, 300 respondents (readers) evaluated 1,000 authors on the four components of communication quality. In addition, readers’ demographics, the length and frequency of social media use, personality, and the linguistic, syntactic, similarity, and sentiment features of the messages were collected. To the best of our knowledge, this is the first study that has attempted to understand an author’s communication quality, which extends the notion of credibility by leveraging human evaluations collected through a more reliable and realistic scenario compared to the case where only a single or manipulated tweet was evaluated for credibility.

We show the influence of the reader’s characteristics on communication quality perceptions. From our study, we found that young and female respondents tended to perceive authors’ communication quality more positively (conversely, old and male respondents tended to show more negative perceptions). In fact, it is hard to generalize the effects of user attributes such as age or gender on the perception of information in social media, as the influence of age and gender on accepting online information varies highly depending on contexts. For example, regarding gender, prior research has generally shown that men trust social media more than women do [48,49]. However, a recent study on understanding the gender difference in social media content shows that women tend to express more trust for posts and messages that consist of anonymous and plain text (https://artios.io/social-media-blind-test-what-happens-when-we-dont-know-who-were-reading/). Regarding age, it seems more studies have shown a negative relationship between age and user perceptions of online and/or social media information. For example, Johnson and Kaye [50] showed a negative relationship between age and perception of online information including social media, and Chung et al. [51] showed a negative relationship between age and perception of online communities. On the other hand, Cassidy [52] presented no strong relationship between age and online news credibility. Since the respondents in our study assessed the authors based on their tweets, our study results may provide additional evidence of a gender effect in the context of evaluating content creators in social media.

We found a strong relationship between the frequency of social media use and communication quality. Frequent use may lead readers to have more critical standpoints toward content and authors in social media. Interestingly, we did not find any strong influence of readers’ personality on communication quality assessment. In general, agreeableness showed positive influences of communication quality. People who have high agreeableness are more likely to consider interpersonal behavior and the quality of social interaction important, and look at things positively [53]. Because of this, in our context, those people are likely to evaluate tweets in a more positive fashion.

We present the relationships between linguistic characteristics of tweets and communication quality. Our results indicate that tweets containing more articles and prepositions, sixltr words, and positive sentiments are more likely to generate readers’ positive perception of authors’ communication quality. Having more articles and prepositions indicate greater formality of the tweets written [41]. Similarly, using sixltr words in the text implies a higher level of education and social class in the text. If the tweet was written in a more positive fashion, it seems that readers also feel positive toward it.

More negations and swear words are likely to generate negative influences. When we consider this finding together with the effect of positive sentiments, the tone of content appears to be important for communication quality assessment. The fact that affect generally makes communication seem more credible is something that one could argue is exactly the opposite of how it “should be” but is in fact quite in line with the nature of persuasion.

For the negative affect, we assume that some readers might find that having more questions and mentions makes the content look busier and does not necessarily give the readers particularly meaningful information. This also relates to our findings in consensus (Table 5), which show high disagreement among the readers if the author’s tweets have more mentions. We also showed the examples of the authors’ tweets that yielded high or low consensus. In addition, tweets similarity showed a strongly negative influence on communication quality assessment. It appears that if a list of the tweets (related to a certain topic and posted by the same author) are similar, readers are likely to give low communication quality to the author. Readers may believe those tweets are spams, because they would look similar and repetitive, which has been found to be one of the characteristics of spam bots [24].

Lastly, using various types of features from profiles, syntactic, linguistics, similarity, and sentiments, we demonstrate the possibility of building accurate models that suggest an author’s level of communication quality of an author based on his or her tweets, with up to 94% accuracy (94% F1 score). As building models using communication quality scores has not been done yet, there is no baseline to directly validate the performance of our models. Nonetheless, if we assume it is fair to compare our models with previous ones, we found that our model performance is reasonably high and is comparable with the scores in previous studies (where they focused on credibility only).

For the model (note that the feature importance for modeling does not necessarily match the results in Table 10), account registration age is the most influential feature. Tweets similarity is the second most influential feature, which highlights the importance of giving readers enough information for better evaluation of social media content. The length of an author’s profile description is found to be important features. This means that authors who describe themselves in more detail are more likely to show their posting behaviors in a more reliable, credible fashion. Previous studies showed that these features had high discrimination powers for classifying spammers and legitimate posters [8,13,17,24,46]; thus, our study results substantiate these characteristics.

Overall, these results demonstrate that a reader’s (human evaluator’s) perceived communication quality of authors can vary depending on the reader’s characteristics. Using human evaluations is a critical step for understanding an author’s communication quality. Therefore, it is important to consider various factors (some of which were identified and measured in our study) to ensure the same level of consistency across evaluators. While it may not be intuitive to use the various reader factors as the features for the models, they can be used during the process of creating training data by assigning a different weight to each reader factor and adjusting the human-labeled values accordingly. For example, for a variable that shows a strong influence on a dependent variable, a weight that will lower its influence can be assigned. The models will then be calibrated based on both the original and the adjusted data, and the performance of the models with each set of data will be analyzed and compared. This process will be repeated until the models have been calibrated by enough samples, and the model that most accurately considers the given context will be chosen. This is one of our research goals for future work.

### Limitations and future work

While our study offers many insights, the study results may not be generalized because we considered only tweets related to the specific keywords. Prior studies indicate that people’s credibility judgment can vary depending on topics [15,16,25]. Because of this, our models may not predict reliable communication quality results for the data generated in other contexts. One of our future plans is to develop models that are reproducible across different topics. In addition, we believe our models could provide better performance by adding more features that are relevant to the author and tweets. For example, research has shown that a user’s personality (not evaluators’ personality) can be identified from the language used in social media [54,55]. Since we have enough tweets for each author, we can also leverage this idea to obtain an author’s personality traits and use them for modeling.

While we tried to use a filtering mechanism to recruit reliable respondents from Amazon Mechanical Turk, we still note a possibility that the survey respondents might want to hide some of their personal characteristics or lie about them. Since this might have happened in our study case, we acknowledge that it could have influenced some of our conclusions and insights.

During data selection, we filtered out authors with less than 10 tweets. We used the tweets that contain a specific keyword (e.g., Olympics, disease outbreaks). The data that we used in the study were somewhat restricted and did not reflect some cases. For example, this may lead to a sample that is biased towards marketers who tend to tweet about a topic fairly frequently, and a smaller percentage of non-marketing but overtly "false or misleading" information. As we indicated previously, our intention to have a specific keyword (topic) in the tweet is that limiting the selection has a benefit for the readers in that the communication quality judgments becomes easier to do since the sets are more easily comparable when topics are related. However, we are planning on collecting survey responses by using any tweets posted by the same author and seeing whether we obtain results and insights similar to the ones in this paper.

## Conclusions

We leverage human evaluations, content analysis, and computational modeling to generate a comprehensive analysis of readers’ evaluations of authors’ communication quality in social media with respect to four factors: author credibility, interpersonal attraction, communication competence, and intent to interact. Our work provides insights for understanding the impact of various reader and content characteristics for the assessment of the author’s communication quality in social media. We demonstrate a possibility of indicating the communication quality of an author. Since the evaluation for the communication quality is highly subjective, a sufficient amount of context and information should be given to evaluators, which will result in better characterizing authors and developing comprehensive and reliable models.
